# Utility of a Laboratory Alert System for Detecting Adverse Drug Reactions in Hospitalised Patients: Hyponatremia and Rhabdomyolysis

**DOI:** 10.3389/fphar.2022.937045

**Published:** 2022-07-06

**Authors:** Mónica Valdés-Garicano, Gina Mejía-Abril, Diana Campodónico, Raúl Parra-Garcés, Francisco Abad-Santos

**Affiliations:** ^1^ Clinical Pharmacology Department, Hospital Universitario de La Princesa, Instituto Teófilo Hernando, Facultad de Medicina, Universidad Autónoma de Madrid (UAM), Instituto de Investigación Sanitaria La Princesa (IP), Madrid, Spain; ^2^ Centro de Investigación Biomédica en Red de Enfermedades Hepáticas y Digestivas (CIBERehd), Instituto de Salud Carlos III, Madrid, Spain

**Keywords:** pharmacovigilance, adverse drug reaction, safety, rhabdomyolysis, hyponatremia

## Abstract

**Background**—Adverse drug reactions (ADRs) are a public health issue, due to their great impact on morbidity, mortality, and economic cost. The use of automatized laboratory alerts could simplify greatly its detection.

**Objectives**—We aimed to evaluate the performance of a laboratory alerts system as a method for detecting ADRs, using hyponatremia and rhabdomyolysis as case studies.

**Methods**—This is a retrospective observational study conducted in 2019 during a 6-month period, including patients hospitalized at the Hospital Universitario de La Princesa. Patients were identified using altered laboratory parameters corresponding to the two signals: “rhabdomyolysis” (creatine phosphokinase >5 times the upper limit of normality (ULN): >1000 U/L for men and >900 U/L for women) and “hyponatremia” (<116 mEq/L) were detected. In cases where ADR was suspected, causality assessment was performed using the algorithm of the Spanish Pharmacovigilance System (SEFV).

**Results**—During the study period, 180 patients were studied for the “rhabdomyolysis” signal, 6 of them were found to have an ADR (3.3%). The sensitivity of the test was 60%, specificity 97%, and positive predictive value 41%. 28 patients were studied for the “hyponatremia” signal, and 11 patients were found to have an ADR (39.3%), with a sensitivity of 76.9%, a specificity of 93.3%, and a positive predictive value of 88.2%. We found no relationship between altered laboratory values and risk of ADR in any of the cases studied.

**Conclusion**—A pharmacovigilance program based on automatized laboratory signals could be an effective method to detect ADR. The study of the “hyponatremia” laboratory alert is more efficient than “rhabdomyolysis”. The evaluation of the hyponatremia alert allows the identification of 12 times more ADRs than the rhabdomyolysis alert, which means less time spent per alert evaluated to identify an ADR.

## Introduction

The World Health Organization (WHO) defines an adverse drug reaction (ADR) as a harmful, unintended reaction to medicines that occur at doses normally used for treatment (WHO, 2002; Safety of Medicines, 2002). ADRs are a frequent cause of illness, disability, or death, and in some countries, they are even among the 10 leading causes of mortality (WHO, 2004).

A meta-analysis of prospective studies conducted in U.S. hospitals by Lazarou et al. (1998) estimated that the overall incidence of serious ADRs in hospitalized patients was around 6.7% and the incidence of deaths from ADRs was about 0.3%. Furthermore, ADR treatment places a largely unrecognized but considerable financial burden on the healthcare system. The need for these additional medical interventions may be avoidable (WHO, 2002). Therefore, mechanisms to assess and monitor the level of safety provided by the clinical use of medicines are essential to prevent or reduce adverse drug effects and improve public health (WHO, 2004).

In hospitals, the most common method used for ADR detection is spontaneous reports. However, this system is subjected to several limitations, notably the existence of a high under-reporting of ADRs (Neubert et al., 2013). Currently, the WHO and the European Medicines Agency (EMA) propose complementing spontaneous reports with specific pharmacovigilance programs to identify drug safety problems as early as possible (Ramirez et al., 2010).

Methods to identify ADRs should be tailored to local needs. In our center, active pharmacovigilance activities include the review of all patients admitted to the hospital but data related to ADRs not apparent at the time of admission or arising during hospitalization are lost. In addition, the diagnosis of ADRs is not always straightforward and tools to facilitate their early identification are part of the strategy to improve patient safety.

In recent years, the availability of computerized databases associated with electronic medical records has made it possible to develop different programs for the detection of ADRs. The methods used by these programs differ between hospitals due to the specific characteristics of each clinical setting (Ramirez et al., 2010). ADR detection systems based on signals generated using laboratory information stand out. Several studies have identified these programs as effective (Levy et al., 1999; Ramirez et al., 2010; Neubert et al., 2006; Dormann et al., 2004; Tegeder et al., 1999; Dormann et al., 2000). In addition, they can be used as a tool for the early detection of ADRs, thereby reducing hospital length of stay and costs caused by ADRs (Dormann et al., 2000). The software developed at our hospital allows the automatic detection of clinically relevant altered analytical values, such as elevation of liver enzymes, amylase, creatine phosphokinase (CK), hematologic alterations and hyponatremia.

The primary research objective of this study was to evaluate the performance of a laboratory alerts system as a method of detecting ADRs, using hyponatremia and rhabdomyolysis as case studies. Secondary objectives were to evaluate the performance of these laboratory signals, estimate the incidence of identified ADRs, and describe the characteristics of patients in whom an ADR has been identified.

## Materials and Methods

### Study Population and Study Design

A retrospective observational study was conducted at the Hospital Universitario de La Princesa, a tertiary level university hospital, including all medical specialties except for pediatrics and gynecology-obstetrics. It has 524 beds and currently covers a population of 323,000 people in Madrid (Basic Information, 2020).

The study population was all patients hospitalized in the hospital during the study period. The study period was 6 months (1 July 2019 to 31 December 2019). These dates were chosen, despite the existence of time periods closer to the study (September 2020–April 2021) to avoid the possible contaminating effect that the SARS-CoV-2 Global Pandemic could have on the validity of the data collected. The data collected were limited to the laboratory signals of “rhabdomyolysis” and “hyponatremia".

The methodology proposed by Ramirez et al. (2010) was used as a reference, with some modifications:

⁃ Definition of the laboratory signals: rhabdomyolysis (value of creatine phosphokinase [CK] >5 times the upper limit of normality (ULN): >1000 U/L for men and >900 U/L for women) and hyponatremia (<116 mEq/L) (Letmaier et al., 2011; Ramírez et al., 2019; Sosa Medellin, 2016; Torres et al., 2015; Arébalo-López et al., 2015).
⁃ Detection of laboratory signals using the “LABORATORY SIGNALS” application developed by the Bioinformatics Department of our hospital.
⁃ Review of medical records when a suspected case was detected. The analysis was not continued in cases whose signal was attributed to the patient’s primary diagnosis or any underlying disease [see Supplementary Annex].
⁃ For the remaining patients, causality assessment was performed using the algorithm developed by the Spanish Pharmacovigilance System (SEFV) (Aguirre and García, 2016). In each patient with suspected ADR, the causality algorithm was applied to each suspected drug by two investigators (MV and GM). Both investigators had clinical experience but one of them had less experience in drug safety assessment. To calculate the sensitivity, specificity and positive predictive value (PPV), the differences in the causality results of the SEFV algorithm of the two evaluators were taken into account. This made it possible to identify some cases in which the result differed and, after discussion of the discrepancies, it was determined whether the alert met ADR criteria or not.
⁃ Suspected adverse reactions that were ultimately not considered as adverse reactions were considered as false positives.
⁃ ADRs detected were reported to the SEFV.

The SEFV algorithm comprises 7 criteria (Aguirre and García, 2016), which are assessed for every drug-ADR pair: 1) Time sequence (chronology between the start of treatment with the suspected drug(s) and the appearance of the adverse effects); 2) Identification of plausible adverse drug reactions using knowledge extracted from the literature; 3) Withdrawal effect: evolution of the adverse effect after withdrawal of the suspected medication; 4) Re-exposure effect: reaction after re-administration of the suspected drug; 5) Alternative explanation for the observed effects; 6) Contributing factors favoring the causal relationship (e.g. renal failure and relative overdose of a drug with predominantly renal elimination); 7) Complementary explorations: serum drug levels, biopsies, positive radiological examinations, positive specific skin tests, etc. The maximum possible score is 12.

Based on the obtained scores, the causal relationship is classified as: unrelated (<1), conditional (1–3), possible (4–5), probable (6–7) and definitive (>7). Only those classified as possible, probable, or defined were considered as drug related.

### Data Analysis

The statistical analysis was accomplished using Microsoft Excel 2021 and the SPSS 22.0 statistical software (SPSS Inc., Chicago, Illinois). The average, standard deviation (SD) and interquartile range (IQR) were calculated for each quantitative variable studied. The incidences of ADRs detected were estimated from the cases with each signal. PPV were calculated for analytical values where possible (primarily by reviewing all data collected, to identify false negatives while minimizing variability). PPV is defined as the number of times an alert is issued with respect to a particular rule and an ADR is confirmed (true positives), divided by the number of times an alert is issued with or without confirmation of an ADR (sum of true positives and false positives) (Handler et al., 2008). The Number of laboratory signals Needed to be Evaluated (NNE) was estimated by determining the number of cases evaluated to detect one ADR. Hypothesis testing for independent samples was performed with SPSS for those variables that were attempted to be correlated in the two groups (age, sex, level of the analytical value, and the possibility of ADR).

### Ethics

The project was approved by the Ethics Committee for Research on Medicines (CEIm) of the Hospital Universitario de La Princesa. As all the information was registered from the electronic medical record without interviewing the patients, it was not necessary to request patients’ informed consent. Researchers respected the confidentiality of every data obtained during the conduct of the study.

## Results

### Rabdomyolysis

In the study of laboratory signal for “rhabdomyolysis”, the “LABORATORY SIGNALS” application detected 388 laboratory alerts from 180 different patients. In 170 patients an alternative cause was found to justify the high CK levels (see Table 1). In 4 of them, no alternative cause was identified but causality with drugs could not be established, and in 6 of them, 1 ADR was detected. No significant differences regarding age, sex, or CK levels were detected between the different groups.

**TABLE 1:** Patients with a rhabdomyolysis alert who met exclusion factors due to underlying diseases that explained the analytical alteration.

	No. (% of Total) N = 180	CK Levels (U/L, Mean ± SD)	Age (Mean ± SD)	Male Sex (No. and %)
Myocardial infarction	58 (32.2)	3.203 ± 2,785	61 ± 14.5	37 (63.8)
Surgery for reasons other than myocardial infarction	24 (13.3)	2.287 ± 1,636	65.8 ± 14.7	16 (66.7)
Surgery due to myocardial infarction	20 (11.1)	2,054 ± 1,084	68.6 ± 9.6	16 (80)
Muscle compression due to a prolonged fall	17 (9.4)	2,943 ± 2,673	82.8 ± 8.1	7 (41.2)
Infections	11 (6.1)	11,231 ± 20,031	73.1 ± 15.3	7 (63.6)
Drug intoxication	10 (5.6)	15,713 ± 29,184	50.5 ± 24.8	6 (60)
Major trauma	8 (4.4)	2,639 ± 2,073	51.3 ± 17.74	8 (100)
Seizures	8 (4.4)	2,623 ± 2,387	40.1 ± 8.8	8 (100)
Acute vascular thrombosis	8 (4.4)	2,506 ± 1,603	77.1 ± 17.1	4 (50)
Extreme physical exercise	4 (2.2)	13,280 ± 16,508	33 ± 11.2	2 (50)
Myositis and other genetic and metabolic disorders	1 (0.6)	3,762	53	1 (100)
Losses from the study	1 (0.6)	7,171	30	1 (100)
Total	170 (94.4)	4,345 ± 9,649	65.4 ± 18.0	113 (66.5)

CK: creatine-phosphokinase enzyme; SD: standard deviation.

The underlying diseases or primary diagnoses that were exclusion criteria for the patients to be studied (Garro Ortiz, 2014; Li et al., 2014; Torres et al., 2015; Deljehier et al., 2018) are detailed in table 1, being myocardial infarction the most common. A patient was lost from the study: it was a foreign patient who returned to his country of origin after the laboratory alteration was detected, with a strong suspicion of suffering from myositis, without any complementary studies.

Therefore, the total number of cases assessed using the SEFV Causality Algorithm was 10, constituting 5.6% of the total of 180 patients with this signal. A possible ADR was found in 6 of them (3.3%), caused by the following drugs: atorvastatin, lorazepam, risperidone, olanzapine, and rapid insulin, which are detailed in Table 2.

**TABLE 2:** Cases of rhabdomyolysis assessed using the SEFV Algorithm.

Category	No. (% of Total) N = 180	Drugs Responsible for ADR	Originating Service	Peak CK Levels (U/L, Mean ± SD)	Age (Mean ± SD)	Sex (%)
No causality was detected, although they did not meet exclusion factors	4 (2.2)	N/A	ED (3)	3,543 ± 2,765	67 ± 23.4	2F (50)
			PSQ			2M (50)
“Possible” causality (score SEFV: 4–5)	4 (2.2)	Rapid insulin	ICU	4,516	43	F
		Lorazepam	ICU	7,518	53	M
		Olanzapine	ED	1,256	59	F
		Risperidone	PSQ	8,337	63	M
“Probable” causality (score SEFV: 6–7)	2 (1.1)	Atorvastatin	IM	6,508	84	M
		Lorazepam	ED	5,282	60	F
“Definitive” causality (score SEFV: ≥8)	0	N/A	N/A	N/A	N/A	N/A
Total patients with ADR	6 (3.3)	N/A	N/A	4,911 ± 2,308	60.3 ± 13.6	3F (50)
						3M (50)
Total number of cases assessed with the SEFV Algorithm	10 (5.6)	N/A	N/A	4,364 ± 2,451	63 ± 17.2	5F (50)
						5M (50)

SD: standard deviation. N/A: Not applicable. ICU: Intensive Care Unit. ED: emergency department. PSQ: psychiatry department. IM: Internal Medicine Department. F: female. M: male.

Most patients experienced a drop in CK levels from the moment it was diagnosed, taking 2–7 days to reach normal values. In patients with ADRs, the drop in CK levels followed drug withdrawal, with a latency period of 1.5 days until the start of its normalization. In addition, 100% of patients with ADR suffered from renal dysfunction due to rhabdomyolysis, which resolved in all cases after the discontinuation of the precipitating agent.

There were 4 false positives, those cases in which a sufficient score in the SEFV algorithm was not achieved after agreement of all investigators, although no alternative cause could be found to justify the CK levels, and there were no false negatives. This second review was understood as gold standard, as it minimizes the variability, in order to make the following calculations: the sensitivity of the test was defined as 60%, the specificity was estimated at 97% and the PPV of the test was over 40.8% (with a confidence level of 95%). The prevalence of this ADR in patients with rhabdomyolysis was 3.3%.

### Hyponatremia

In the case of ADRs due to “hyponatremia”, the “LABORATORY SIGNALS” application detected 50 laboratory levels with concentration of sodium (Na) in blood serum below 116 mEq/L, in 28 different patients. There were 11 confirmed ADR cases (39.3%). In 17 patients (60.7%) ADR was excluded as an alternative cause of hyponatremia was present (see Table 3). No significant differences regarding age, sex, or hyponatremia levels were found between these groups of patients.

**TABLE 3:** Patients with hyponatremia who met exclusion factors due to underlying diseases that explained the analytical alteration.

	No. (% of Total) N = 28	Na Levels (mEq/L, Mean ± SD)	Age (Mean ± SD)	Female Sex (No. and %)
Diarrhea and other gastrointestinal disorders	5 (17.9)	110.6 ± 4.6	72.6 ± 14.3	4 (80)
Major surgeries	4 (14.3)	111.3 ± 5.8	66.3 ± 15.8	2 (50)
Congestive heart failure^a^	3 (10.7)	107.1 ± 7.0	89.3 ± 8.0	2 (66.7)
Potomania	3 (10.7)	114.7 ± 0.6	76 ± 19	2 (66.7)
Liver cirrhosis	1 (3.6)	115	58	0
Pneumonia	1 (3.6)	116	49	1 (100)
Total	17 (60.7)	111.4 ± 5.1	72.4 ± 16.2	11 (64.7)

Na: sodium. mEq/l: milliequivalents per liter. SD: standard deviation.

a Heart failure was considered an alternative explanation of ADR, because it affects the ability to excrete ingested water by increasing antidiuretic hormone levels and is therefore a cause of hyponatremia.

The underlying diseases or primary diagnoses that were excluded (Letmaier et al., 2011; Ramírez et al., 2019), are listed in Table 3, being the most common diarrhea.

Regarding the drugs causing the ADRs: 18 drugs were found that met causality criteria to be defined as ADRs, in 11 different patients. The demographic characteristics of each patient and their clinical service and the assessment of causality of the SEFV algorithm for each drug, are shown in Table 4. In 7 of the 11 cases, it was not possible to determine the drug causing the ADR because there were 2 drugs that could be responsible, either because of concomitant administration of both separately, or because the pharmaceutical presentation included both, in which case the label of the combined drug was evaluated. These cases of combined administration of two drugs in a single tablet were: trimethoprim-sulfamethoxazole, amiloride-hydrochlorothiazide, and losartan-hydrochlorothiazide. It is noteworthy that 8 of the 11 drugs involved were diuretics.

**TABLE 4:** *Cases of hyponatremia assessed using the SEFV Algorithm*.

Category	No. (% of Total) N = 28	Drugs Responsible for ADR		Clinical Service	Na Levels (mEq/L)	Age	Sex (%)
		Drug 1	Drug 2				
“Possible” causality (score SEFV: 4–5)	8 (28.6)	Chlorthalidone	Enalapril	ED	114	58	F
		Hydrochlorothiazide	Amiloride	ED	111	94	F
		Hydrochlorothiazide	Enalapril	REU	114.8	85	F
		Hydrochlorothiazide	Losartan	ED	115.3	83	F
		Hydrochlorothiazide	N/A	ED	116	84	M
		Furosemide	N/A	ED	116	75	F
		Mirtazapine	Gabapentin	IM	115	86	F
		Trimethoprim	Sulfamethoxazole	ED	102	53	M
“Probable” causality (score SEFV: 6–7)	2 (7.14)	Furosemide	N/A	ED	115.6	97	F
		Methotrexate	N/A	ED	116	19	F
“Definitive” causality (score SEFV: ≥8)	1 (3.57)	Furosemide	Spironolactone	ED	109	61	F
Total with sufficient score SEFV ≥4	11 (39.28)	N/A	N/A	N/A	113.2 ± 4.3	72.3 ± 22.9	9F (81.8)
							2M (18.2)

N/A: Not applicable. ED: Emergency Department. REU: Rheumatology Department. IM: Internal Medicine Department. F: female. M: male.

Treatment was required in 92.9% of the patients studied. Hyponatremia was corrected within 1–3 days in 89.3% of cases, lasting up to 5–7 days in 3 cases. Two of the patients (none with suspected ADRs) died during the episode (both due to sepsis). In the case of ADRs, the recovery of the analytical value after drug withdrawal did not require more than 2 days in any case.

After a subsequent review of the results by a second investigator, three false positives (in which an alternative cause was finally found, therefore they are represented in Table 3) and one false negative were found. After the relevant calculations, the sensitivity of the test was defined as 76.9%, the specificity as 93.3%, and the PPV of the test as 88.2%. The prevalence of ADRs in patients with hyponatremia was 39.2%.

### Comparison of the Two Signals

During 2019 there were 15,898 admissions at our hospital, so the annual incidence of these ADRs is 75.5 cases of rhabdomyolysis per 100,000 admissions and 138.4 cases of hyponatremia per 100,000 admissions. With respect to the parameter “Number of laboratory signals Needed to be Evaluated” (NNE), 30 cases (180/6) need to be reviewed to find an ADR in the case of the laboratory signal “rhabdomyolysis”, and 2.5 cases (28/11) in the case of the signal “hyponatremia”. Therefore, the evaluation of the hyponatremia alert allows the identification of 12 times more ADRs than the rhabdomyolysis alert, which means less time spent per alert evaluated to identify an ADR.

## Discussion

The prevalence of possible ADRs found when studying the analytical signal of “rhabdomyolysis” was 3.3%, a result similar to that found in other similar studies (Haerian et al., 2012). Female sex is a risk factor for suffering ADRs (Rubio Mirón and Sánchez Rubio, 2008), and in the present study there was a marked increase in the proportion of women over men in the group with ADRs versus those with non-drug-related CK elevation (50% compared to 33%), although no significant differences were found (which would be expected to be found if the sample size were larger). Nor was it possible to find significant differences in age or a correlation between CK levels and the likelihood of ADR.

As for the drugs causing ADRs, all of them were reported in the literature to cause rhabdomyolysis as a side effect (Rubio Mirón and Sánchez Rubio, 2008; Oshima, 2011; Arébalo-López et al., 2015; Torres et al., 2015). Only three of them listed rhabdomyolysis as an adverse effect in the corresponding drug label: risperidone, atorvastatin, and olanzapine. The remaining 2 drugs involved (lorazepam and rapid insulin) do not mention rhabdomyolysis as an adverse effect, but this is explained by the much lower frequency of these adverse reactions in these cases (Oshima, 2011; Haerian et al., 2012; Torres et al., 2015). This leads us to believe that more active pharmacovigilance could provide data that would allow these ADRs to be better characterized, possibly including rhabdomyolysis as an adverse reaction in the drug label in the future.

It is noteworthy that, although statins are usually the most frequent pharmacological group causing rhabdomyolysis (Oshima, 2011; Garro Ortiz, 2014; Torres et al., 2015), in our study only 1 of the 6 drugs found was a statin. However, about 60% of patients who suffered a myocardial infarction, underwent surgery, or suffered a muscle compression due to a fall, were on statin treatment. All these patients had some underlying disease that explained the CK elevation, which could in some cases mask an ADR.

Regarding the hyponatremia signal: based on the results obtained (prevalence close to 40%, sensitivity of 76%, specificity of 93%, and PPV of 88%), there is a high correlation between the patients studied and patients who truly present an ADR, demonstrating the usefulness of its routine study. In this case, no significant differences were found in the age, sex, or sodium levels of patients with ADRs compared to those with hyponatremia produced by alternative causes.

Of the drugs that met the causality criteria for ADR, 72.2% were diuretics, as it is one of the most frequent drug groups that causes hyponatremia as ADR (Ramirez et al., 2010; Spasovski et al., 2014). However, diuretics are part of the therapeutic strategies used in heart failure, which is in itself a cause of hyponatremia, so there is a confounding parameter in the assessment of this ADR.

Of the drugs involved, only methotrexate did not have hyponatremia as a possible adverse effect listed on the drug label but it was reported in the literature (Liamis et al., 2016; Spasovski et al., 2014). Additionally, 6 cases were found that appeared to be due to a drug-drug interaction. In all cases, each drug was described as a potential cause of hyponatremia on its own, thus ruling out the possibility that the ADR only occurred in the case of interaction, and in any case, raises a possible potentiation of the ADR. The drug combinations were trimethoprim-sulfamethoxazole, furosemide and spironolactone, hydrochlorothiazide and amiloride, chlorthalidone and enalapril, enalapril and hydrochlorothiazide, and mirtazapine and gabapentin.

The drugs associated with the two ADRs evaluated are different, as is their prevalence of use in the general population. In addition, more drugs are associated with hyponatremia than with rhabdomyolysis. Nevertheless, the aim of this work is to evaluate the possibility of detecting ADRs using the laboratory’s alert program.

If we consider the approximation of “NNE” that was calculated (it is necessary to review 30 patients with rhabdomyolysis to find an ADR, and 2.5 patients with hyponatremia to find an ADR), the study of the laboratory signal “hyponatremia” is more efficient than that of “rhabdomyolysis” for detecting ADRs. Moreover, it is also simpler: for the “hyponatremia” signal, 90% (10/11) of ADRs included in the discharge report a statement regarding the pharmacological origin of the alteration, compared to 16% (1/6) in the case of “rhabdomyolysis”. This could indicate that there is greater awareness among medical staff on the possibility of ADRs in the case of hyponatremia, raising the possibility of studying whether there is an association between the best-known adverse reactions with better treatment, and with faster recovery and lower morbidity and mortality for the patient. One could even consider the need for further training of physicians in these issues so that they can suspect less prevalent and less known adverse reactions.

In terms of limitations of the study, as the data were not compared to a gold standard, the sensitivity and specificity of the signals were calculated by comparing a first and second review of the data. About the causality algorithm application, it is necessary to highlight that there is no internationally validated algorithm and that the algorithm of SEFV is only one tool for evaluation. These algorithms depend closely on the physician’s clinical experience. There were several discrepancies between the results of the two evaluators, so the PPV for the both laboratory signals studied were very low. However, we believe that this measure can be improved with specific training and experience. In this regard, we recommend clinicians to keep up to date with drug safety surveillance, which will allow early identification and treatment of ADRs.

It is important to consider that the incidence of ADRs is low, that resources to evaluate alerts are limited and that we are interested in detecting a greater number of ADRs with the least possible effort. Therefore, specificity has been prioritized over sensitivity of the method. The cut-off points have been established to rule out mild cases, detect serious ADRs and obtain a manageable number of alerts to evaluate. Although this may be a limitation of the study, being less restrictive in the evaluation cut-off points could generate a lot of noise, making it difficult to identify and manage ADRs in a timely manner.

For most borderline cases, it would have been necessary to re-expose the patient to the drug to conclude causality between the altered laboratory results and a possible ADR. The ethical aspects of this measure should be taken into consideration, as it is not a simple decision to expose the patient to a potentially harmful medication, without any other clinical or therapeutic reason to justify the re-exposure. This aspect should be taken into account in future studies as a limitation.

As this was a retrospective analysis, the information contained in the medical records was sometimes incomplete and it was not possible to contact the patient or specialist to obtain additional information.

Although the population attended in our hospital during the study period was approximately 8,000 patients, a series of limitations have risen such as the selection bias that might have occurred when limiting the study to 6 months in a single hospital in Madrid. As a consequence, the data obtained could only be extrapolated to a population with similar characteristics.

## Conclusions

A pharmacovigilance program based on automatized laboratory signals could be an effective method to detect ADRs in hospitalized patients. The Causality Algorithm of the Spanish Pharmacovigilance System is suitable for this purpose. The application used allows to identify ADRs and to help clinicians in the specific management of the ADR if required.

The study of the laboratory signal “hyponatremia” is more efficient than that of the signal “rhabdomyolysis”, as it requires a smaller number of cases to be examined to find an ADR. The prevalence of ADR found for each of the signals is 39.3% for “hyponatremia” and 3.3% for “rhabdomyolysis”. In neither case has it been possible to establish a relationship between the magnitude of the alteration in the laboratory value and the possibility that it was caused by drugs.

The study of adverse drug reactions using automatized laboratory signals can be very useful to obtain information that may be missed during the clinical assessment. To be able to do this properly, healthcare professionals must be meticulous when completing a patient’s clinical history, avoiding missing data that could be useful afterwards.

Knowledge about the potential for a drug to cause a particular adverse reaction makes it easier to recognize, resulting in optimal treatment for the patient.

It is important to continue active pharmacovigilance to collect more information on adverse drug reactions, as the less frequent ones may still be largely unknown.

In addition, pharmacovigilance activities include the notification of these ADRs to the SEFV, which groups and evaluates ADR notifications from all over the country with the aim of identifying new risks derived from the use of drugs. Thus, optimizing the reporting activity indirectly leads to improvements in the safe use of the drugs and in the health of the population.

## Data Availability

The raw data supporting the conclusions of this article will be made available by the authors, without undue reservation.
