# Supplementary material for: Utility of a Laboratory Alert System for Detecting Adverse Drug Reactions in Hospitalised Patients: Hyponatremia and Rhabdomyolysis
Source: Front Pharmacol. 2022 Jul 6;13:937045. doi: 10.3389/fphar.2022.937045 (PMC9299062; doi:10.3389/fphar.2022.937045)
Supplement: Supplementary file 1 [file DataSheet1.pdf]

**SUPPLEMENTARY ANNEX**

***Alternative causes that rule out the suspicion of drug-induced rhabdomyolysis***
(Deljehier et al, 2018; Torres et al, 2015; Garro Ortiz et al, 2014; Li Y et al, 2014)

Electric current accidents and burns to a significant proportion of the body.

Electrolyte abnormalities: hypocalcaemia, hypophosphataemia, hypokalaemia, hypo/hypernatraemia.

Changes in body temperature: malignant hyperthermia, neuroleptic malignant syndrome, hypothermia.

Prolonged surgery.

Seizures.

Genetic defects: disorders of glycolysis or glycogenolysis (McArdle's disease, Tarui's disease, LDH deficiency, etc.), lipid metabolism disorders, Duchenne and Becker myotonic dystrophies, Krebs cycle disorders, mitochondrial diseases, G6PDH deficiency, etc.

Drugs of abuse (alcohol, cocaine, amphetamines, etc.).

Extreme physical exercise.

Autoimmune diseases: myositis, dermatomyositis, polymyositis.

Muscle hypoxia: acute vascular thrombosis, vessel clamping during surgery, myocardial infarction.

Infections: Influenza A and B, Coxsackie virus, Epstein-Barr virus, HIV in window period, Legionella, Herpes virus, Salmonella, Streptococcus pyogenes, Staphylococcus aureus, tularemia, Clostridium, sepsis.

Intoxications: carbon monoxide, biological toxins.

Endocrine disorders: hyperaldosteronism, hypothyroidism, diabetic ketoacidosis.

Trauma and muscle compression (car accidents, prolonged falls).

*G6PDH: glucose-6-phosphate dehydrogenase; HIV: human immunodeficiency virus; LDH: lactate dehydrogenase.*

***Alternative causes that rule out suspected drug-induced hyponatremia***
(Ramírez et al, 2019; Letmaier et al, 2016)

Major abdominal or thoracic surgery, pituitary surgery, postoperative pain.

Liver cirrhosis.

Hereditary diseases: gain-of-function mutations of the vasopressin V2 receptor.

Pulmonary diseases: asthma, cystic fibrosis, respiratory failure associated with positive pressure breathing.

Major haemorrhages.

Infections: bacterial or viral pneumonia, lung abscess, tuberculosis, aspergillosis, encephalitis, meningitis, brain abscess, Rocky Mountain fever, malaria.

Congestive heart failure.

Advanced renal failure.

Neoplasms: carcinoma of lung, oropharynx, gastrointestinal (stomach, pancreas), genitourinary (ureter, bladder, prostate, endometrium), endocrine thymoma, lymphomas, sarcomas (Edwing's sarcoma), olfactory neuroblastoma.

Gastrointestinal losses (diarrhoea).

Neurological disorders: subdural haematoma, subarachnoid haemorrhage, stroke, brain tumour, head injury, hydrocephalus, cavernous sinus thrombosis, multiple sclerosis, Guillain-Barré syndrome, Shy-Drager syndrome, Delirium Tremens, acute intermittent porphyria.

Psychiatric disorders: potomania (primary polydipsia).
